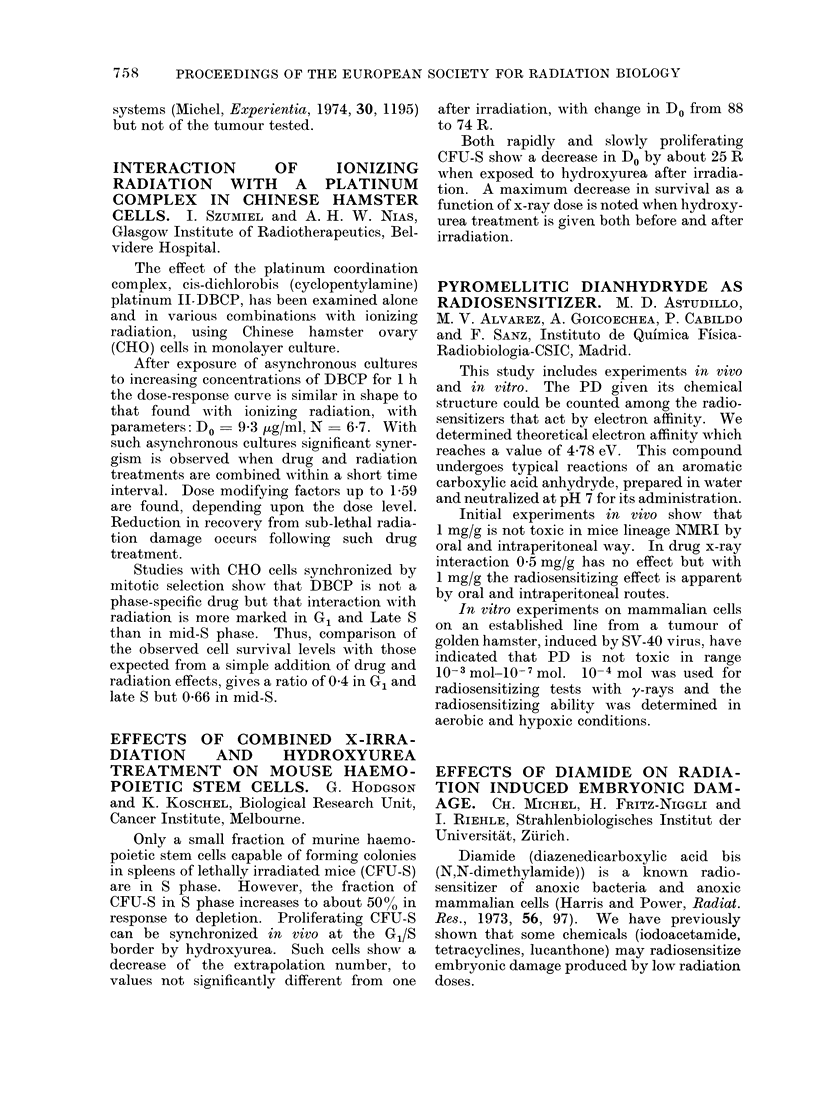# Proceedings: Pyromellitic dianhydryde as radiosensitizer.

**DOI:** 10.1038/bjc.1975.313

**Published:** 1975-12

**Authors:** M. D. Astudillo, M. V. Alvarez, A. Goicoechea, P. Cabildo, F. Sanz


**PYROMELLITIC DIANHYDRYDE AS RADIOSENSITIZER.** M. D. ASTUDILLO, M. V. ALVAREZ, A. GOICOECHEA, P. CABILDO and F. SANZ, Instituto de Química Física-Radiobiologia-CSIC, Madrid.

This study includes experiments *in vivo* and *in vitro*. The PD given its chemical structure could be counted among the radiosensitizers that act by electron affinity. We determined theoretical electron affinity which reaches a value of 4·78 eV. This compound undergoes typical reactions of an aromatic carboxylic acid anhydryde, prepared in water and neutralized at pH 7 for its administration.

Initial experiments *in vivo* show that 1 mg/g is not toxic in mice lineage NMRI by oral and intraperitoneal way. In drug x-ray interaction 0·5 mg/g has no effect but with 1 mg/g the radiosensitizing effect is apparent by oral and intraperitoneal routes.

*In vitro* experiments on mammalian cells on an established line from a tumour of golden hamster, induced by SV-40 virus, have indicated that PD is not toxic in range $10^{-3}$ mol–$10^{-7}$ mol. $10^{-4}$ mol was used for radiosensitizing tests with $\gamma$-rays and the radiosensitizing ability was determined in aerobic and hypoxic conditions.